# Identification of fusarium head blight resistance markers in a genome-wide association study of CIMMYT spring synthetic hexaploid derived wheat lines

**DOI:** 10.1186/s12870-023-04306-8

**Published:** 2023-05-31

**Authors:** Mitra Serajazari, Davoud Torkamaneh, Emily Gordon, Elizabeth Lee, Helen Booker, Karl Peter Pauls, Alireza Navabi

**Affiliations:** 1 Department of Plant Agriculture, University of Guelph, Guelph, ON N1G 2W1 Canada; 2 Département de Phytologie, Université Laval, Québec City, Québec G1V 0A6 Canada; 3 Institut de Biologie Intégrative Et Des Systèmes (IBIS), Université Laval, Québec City, Québec G1V 0A6 Canada

**Keywords:** Deoxynivalenol, Fusarium head blight, GWAS, Resistance, SNP marker, Synthetic hexaploid derived wheat

## Abstract

Fusarium head blight (FHB), caused by *Fusarium graminearum*, is one of the most destructive wheat diseases worldwide. FHB infection can dramatically reduce grain yield and quality due to mycotoxins contamination. Wheat resistance to FHB is quantitatively inherited and many low-effect quantitative trait loci (QTL) have been mapped in the wheat genome. Synthetic hexaploid wheat (SHW) represents a novel source of FHB resistance derived from *Aegilops tauschii* and *Triticum turgidum* that can be transferred into common wheat (*T. aestivum*). In this study, a panel of 194 spring Synthetic Hexaploid Derived Wheat (SHDW) lines from the International Maize and Wheat Improvement Center (CIMMYT) was evaluated for FHB response under field conditions over three years (2017–2019). A significant phenotypic variation was found for disease incidence, severity, index, number of Fusarium Damaged Kernels (FDKs), and deoxynivalenol (DON) content. Further, 11 accessions displayed < 10 ppm DON in 2017 and 2019. Genotyping of the SHDW panel using a 90 K Single Nucleotide Polymorphism (SNP) chip array revealed 31 K polymorphic SNPs with a minor allele frequency (MAF) > 5%, which were used for a Genome-Wide Association Study (GWAS) of FHB resistance. A total of 52 significant marker-trait associations for FHB resistance were identified. These included 5 for DON content, 13 for the percentage of FDKs, 11 for the FHB index, 3 for disease incidence, and 20 for disease severity. A survey of genes associated with the markers identified 395 candidate genes that may be involved in FHB resistance. Collectively, our results strongly support the view that utilization of synthetic hexaploid wheat in wheat breeding would enhance diversity and introduce new sources of resistance against FHB into the common wheat gene pool. Further, validated SNP markers associated with FHB resistance may facilitate the screening of wheat populations for FHB resistance.

**Supplementary Information:**

The online version contains supplementary material available at 10.1186/s12870-023-04306-8.

## Background

Cereal crops have played a major role in shaping societies and providing food, feed, and raw materials for industrial uses. Food security, however, is compromised by a growing human population, shortages of water and nutrients, as well as biotic and abiotic stresses. Worldwide, 400 million people faced food insecurity during 2015–2019 [1]. Breeding for high-yielding and stress-tolerant crops is indispensable for mitigating the global food crises [1]. In recent years, multi-omics approaches including phenomics, genomics, and proteomics have accelerated plant selection and enabled a faster and more accurate molecular breeding process [2]. Allopolyploid wheat is the most important cereal grain for human food and animal feed mainly due to its adaptability to diverse environments worldwide [3].

Recently, the annual increase in wheat yields has slowed down [4]. In the United Kingdom, however, the average wheat yield has remained unchanged at eight t.hm^−2^ for 12 years [4, 5]. This stagnation in yield gains may be due to reductions in wheat genetic diversity [4]. In addition, fungal diseases such as FHB are major yield-reducing factors. FHB not only causes yield losses but also produces mycotoxins, which reduce grain quality and pose significant risks to animal and human health [6].

Among *Fusarium* species that cause FHB, *F. graminearum* is the predominant species in North America that produces deoxynivalenol (DON) as a secondary metabolite [6]. FHB was first reported in Canada in 1919 [7] and FHB epidemics have caused significant economic losses across Canada since 1980 [7]. Breeding for resistance against FHB is complicated by different types of resistance mechanisms. These include Type I (resistance to initial infection), Type II (resistance to the spread of symptoms in the spike), Type III (resistance to accumulation of mycotoxins), Type IV (resistance to kernel infection), and Type V (resistance to yield loss) [8–11].

The development of FHB resistance varieties, which is widely regarded as the most effective way of controlling FHB, requires the incorporation of diverse sources of resistance into breeding programs. To increase the diversity in the bread wheat gene pool, CIMMYT has developed thousands of synthetic hexaploid wheat (SHW) accessions from crosses between tetraploid durum wheat (*Triticum turgidum*, AABB) and diploid wild goat grass (*Aegilops tauschii*, DD) [12]. In order to conquer unfavorable characteristics of SHW lines such as late maturity, height, and being hard to thresh, breeding programs use the backcrossing method to the common wheat lines and create synthetic hexaploid derived wheat (SHDW) lines [13].

Incorporation of the D genome from *Aegilops tauschii* into SHWs resulted in diverse populations with resistance or tolerance to environmental stresses including resistance to stripe rust and Septoria leaf blotch [12]. Wild wheat relatives and synthetic hexaploids, however, have not been used as sources of resistance to Fusarium in wheat breeding programs mainly due to rachis shattering tendency [14, 15]. Although durum wheat is susceptible to FHB, introgression of resistance from hexaploid wheat improved resistance to FHB in some durum wheat lines [14, 16]. The FHB resistance, however, may be compromised by a suppressor in durum wheat [17]. On the other hand, the D genome, which does not exist in durum wheat, may be involved in the FHB resistance [16, 17]. Incorporation of the D genome from *A. tauschii* into an SHW population improved resistance against FHB by reducing disease severity (18.3%) compared with tetraploid counterparts [16].

Resistance to FHB is a quantitative trait with a complex nature. Since the first QTL study of FHB resistance in 1999 [18], approximately 500 QTL have been identified with only 20% having a major effect on FHB resistance [19]. Also, several GWAS have been conducted using single nucleotide repeat (SSR) or sequence-tagged site (STS) markers to study FHB resistance [19–22]. Recently, 9 K and 90 K SNP chip arrays were shown to be more effective in identifying FHB resistance QTL compared with SSR markers [19].

The majority of FHB resistance QTL are population-specific and non-stable in different environments. In contrast, *Fhb1* from Chinese germplasm is stable in different wheat backgrounds and environments without a negative effect on yield [19, 23, 24]. The Asian landraces such as Sumai 3, Wangshui bai, and their derivatives, which contain *Fhb1*, *Fhb2*, *Fhb4*, *Fhb5*, and *Qfhs.nau-2B* QTL, are the most important sources of FHB resistance worldwide [18, 19, 25, 26]. The QTL from Sumai 3 have been incorporated into more than 20 spring wheat cultivars, which have been released since 1999 in the northern United States and Canada [19]. In addition, Alsen and ND744 from North Dakota were used as bridges to introduce Sumai 3 FHB resistance to Canadian wheat varieties such as AAC Brandon, AAC Elie, Cardale, AC Carberry, and CDC VR Morris. The winter wheat lines 25R18, 25R42, and 25R51 from Pioneer, which contain Sumai 3 FHB resistance QTL, have been incorporated into winter wheat breeding programs in Ontario [24].

The main goals of this study were to evaluate FHB resistance in an SHDW spring wheat population, identify new sources of resistance to FHB, determine SNP markers associated with FHB resistance, and identify FHB resistance candidate genes.

## Results

### Evaluation of field and post-harvest FHB traits

**Fig. 1:**
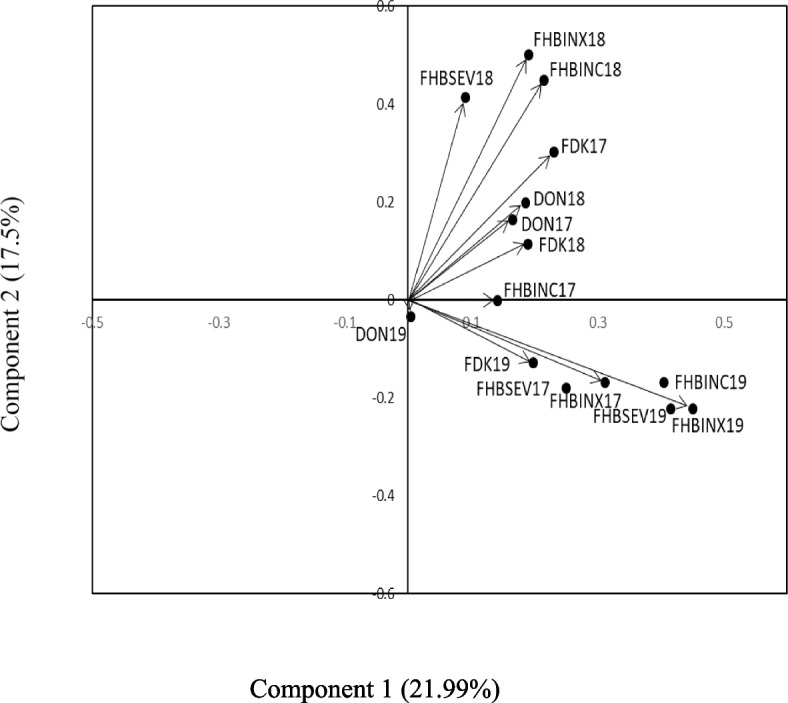
A bi-plot analysis of SHDW lines for FHB traits across three years (2017–2019)

An evaluation of 194 SHDW for FHB symptoms was conducted for three years (2017–2019) under artificial inoculation. These evaluations revealed variable results for FHB field traits (incidence, severity, and index) and post-harvest traits (FDKs number and DON content) among wheat lines (Table 1). Although FHB field and post-harvest traits were significantly different among the wheat lines in 2017, no significant differences among the field traits were found in 2018. Post-harvest traits, however, were significantly different. Similar to 2017 results, significant differences among wheat lines for all field traits and post-harvest traits were found in 2019 except for DON content. Bi-plot (Fig. 1) and correlation analyses (Additional Fig. 1) revealed positive relationships between DON content and other FHB traits except for FHB incidence, FDKs, and severity in 2017. DON content and other FHB traits were positively correlated in 2018. DON content, however, was not correlated with other FHB traits in 2019. In all years, the FHB index was positively correlated with other FHB traits except for DON content in 2019 (Additional Fig. 1). The relatively low mean values for the FHB traits in 2018 were indicative of a low FHB pressure (Table 1). The disease index in 2% and 15% of the SHDW lines was lower than AC Carberry (moderately susceptible) in 2017 and 2019, respectively. In both years, the disease index in 3% and 8% of the SHDW lines was < 11. This index was 11–30 for 46% of the lines in 2017 and 42% of the lines in 2019. Among 194 SHDW lines, 11 individuals showed below 10 ppm DON content in both 2017 and 2019 (Additional Table 2), and 51% and 50% of the lines showed a disease index of > 30 in 2017 and 2019, respectively (data not shown). The interaction analysis revealed a significant interaction between year and genotype for 2017, 2019, and all three years (Table 1).

**Table 1:** Significant differences among SHDW lines for FHB field and post-harvest traits

**Year**	**Traits**	**FHBINC**	**FHBSEV**	**FHBINX**	**FDK**	**DON**
2017	*P* value	0.0265	< .0001	0.0002	< .0001	< .0001
	Mean	66.49	47.03	31.25	2.84	12.8
	AAC Scotia	60	33	19.8	0.5	4.8235
	Carberry	70	14	9.8	0	0.7865
	Hoffman	52.5	50	26.25	0	4.073
	Norwell	40	41.5	16.6	0.5	1.5635
	Sable	80	33	26.4	0	2.237
	Pasteur	72.5	41.5	29.875	0.5	5.465
2018	*P* value	0.0656	0.636	0.4035	0.0005	< .0001
	StdErr	8.38	9.81	1.7	1.14	0.86
	Mean	7.26	6.91	1.15	1.58	1.43
	AAC Scotia	0	0	0	0	0.0455
	Carberry	0	0	0	1	0.0525
	Hoffman	5	3.5	0.35	0.5	0.2685
	Norwell	15	3.5	1.05	1	0.1155
	Sable	15	10.5	1.4	0	0.1455
	Pasteur	5	3.5	0.35	0	0.068
2019	*P* value	< .0001	< .0001	< .0001	< .0001	0.0902
	StdErr	12.8	10.95	10.56	4.16	1.97
	Mean	63.83	45.81	31.54	28.72	10.43
	AAC Scotia	27.5	32	9.25	23.5	5.87
	Carberry	57.5	43.5	31.2	18.25	10.02
	Hoffman	47.5	33	15.675	23.5	7.95
	Norwell	45	33	14.85	20	6.785
	Sable	82.5	64.5	53.575	19.75	8.32
	Pasteur	55	17.5	8.575	24.5	6.955
2017 and 2019	*P* value	0.2151	0.0019	0.0002	0.2587	0.2254
	StdErr	10.21	9.59	8.95	13.31	3.7361
	G × Y*	0.0006	0.0027	0.0012	< .0001	< .0001
	Mean	65.16	46.42	31.39	15.8	11.74
2017–2019	*P* value	0.107	0.1304	0.0017	0.026	0.2254
	StdErr	20.66	15.16	4.16	9.09	3.73
	G × Y*	< .0001	< .0001	< .0001	< .0001	< .0001
	Mean	45.86	3.25	21.31	11.05	8.3

*DON* Deoxynivalenol, *FDKs* Fusarium damaged kernels, *FHBINC* Incidence, *FHBINX* Index, *FHBSEV* Severity, *G* × Y* Genotype by year interaction, *StdErr* Standard error

### Genotyping and population genetic analyses

**Fig. 2:**
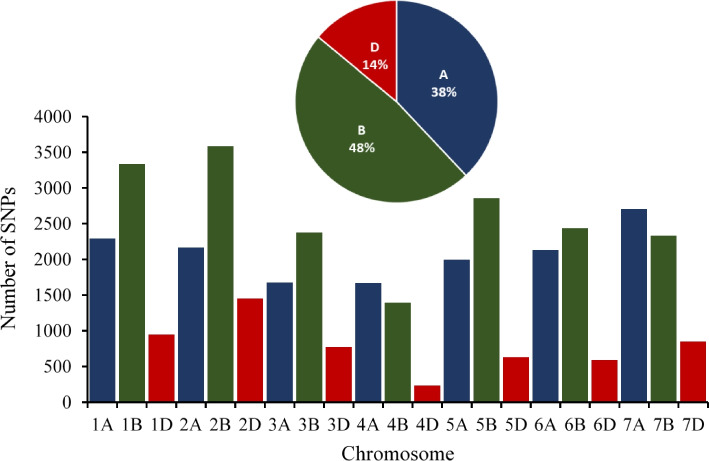
Number of polymorphic SNPs on each chromosome in the SHDW panel

**Fig. 3:**
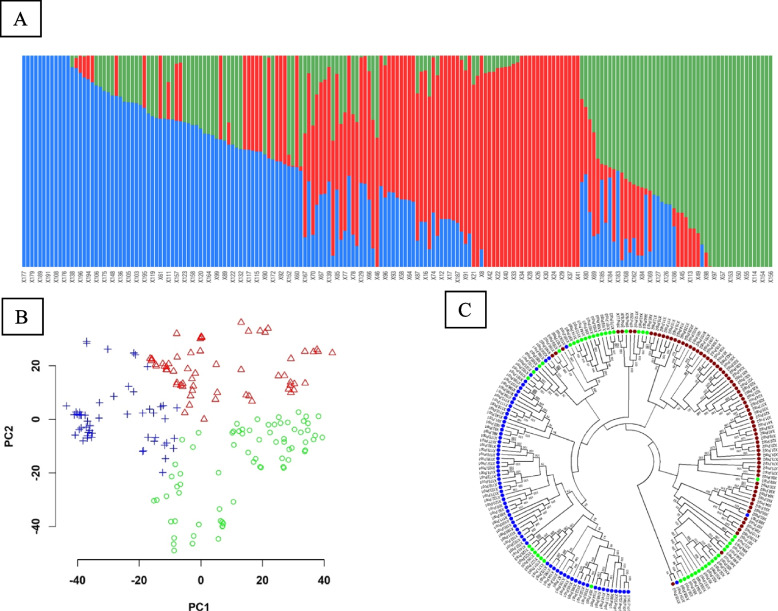
Genetic structure of the SHDW panel. A and B) fastStructure analysis [27] of SNP diversity in the SHW panel based on a ChooseK analysis of the number of subpopulations. The three subpopulations are depicted in blue, red, and green. C) A neighbor-joining phylogenetic tree [28] was constructed in MEGA7 [29] and assessed by bootstrapping (1,000 X) [30]

To obtain genome-wide nucleotide variants, all 200 wheat accessions (194 spring SHDW from CIMMYT and 6 check cultivars) were genotyped using Illumina’s iSelect 90 K SNP chip. Genotypic data were filtered for missing data > 10%, MAF < 5%, and heterozygosity > 50%, which resulted in 31 K high-quality SNPs. The missing data were then imputed. Of these variants, 14,622, 18,299, and 5470 SNPs were located in A, B, and D genomes, respectively (Fig. 2). The 31 K SNP panel was used for population genetic analysis. The PCA, phylogenetic tree clustering, and population structure analyses suggested that the SHDW panel was composed of three (K = 3) sub-populations (Fig. 3 and Additional Fig. 2). A genome-wide linkage disequilibrium (LD) analysis showed a mean LD decay of 200 kb at r^2^ < 0.2, which was comparable to previous studies (Additional Fig. 3).

### Genome-wide association analysis

**Fig. 4:**
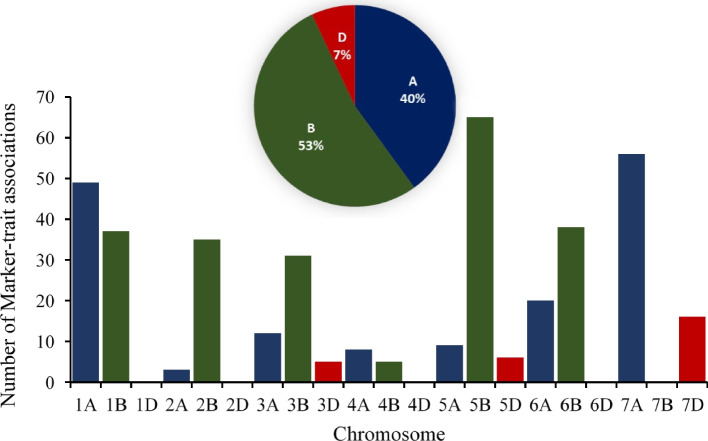
Number of markers associated with FHB resistance traits on each chromosome in the SHDW panel

A GWAS analysis was performed with the population structure (P) and cryptic relatedness (K*) as covariates to reduce false positive signals. Using this approach, 52 significant marker-trait associations (MTAs) for FHB resistance were identified (Additional Table 1). These traits consisted of DON content, percentage of FDKs, FHB index, disease incidence, and disease severity. For DON content, five MTAs were found on chromosomes 2B, 3B, 4B, 6B, and 7A. For percentage of FDKs, thirteen MTAs were found on chromosomes 2B, 3B, 5A, 7D, 1A, 4A, 7A, 1B, 2B, 3B, 5B, and 3D. For the FHB index, eleven MTAs were found on chromosomes 5A, 6A, 1B, 6B, 2A, 6A, 2B, 5B, and 7D. For disease incidence, three MTAs were found on chromosomes 1A and 6B. For disease severity, twenty MTAs were found on chromosomes 1A, 3A, 7A, 1B, 2B, 3B, 5B, 6B, and 5D (Fig. 4 and Additional Fig. 4A-E).

**Fig. 5:**
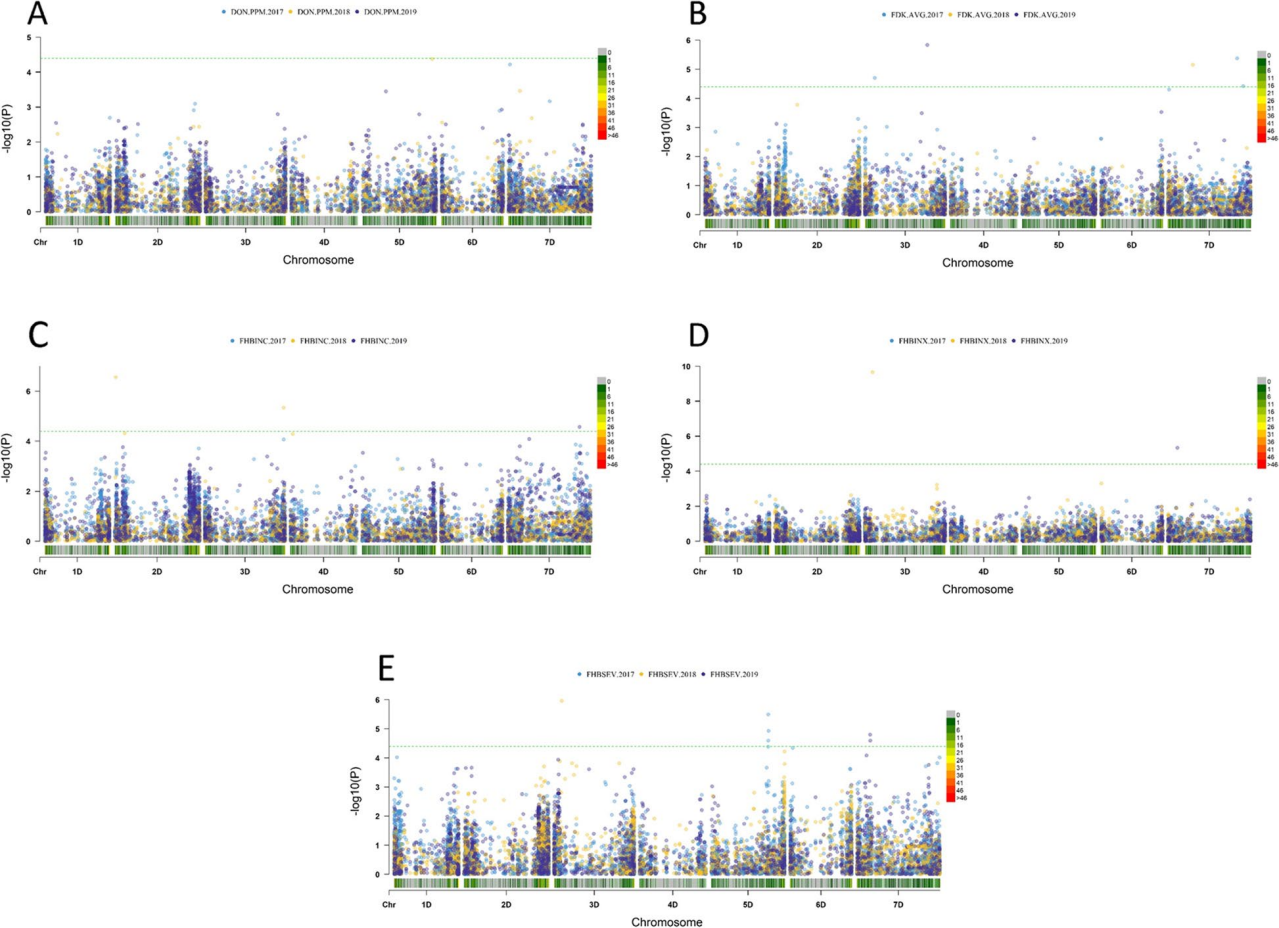
Manhattan plots of associations between SNPs and FHB traits of D genome in the SHW panel across three years (2017–2019). **A** Deoxynivalenol content DON ppm, **B** The average of Fusarium Damaged Kernels FDKave, **C** Fusarium Head Blight Incidence FHBINC, **D **Fusarium Head Blight Index FHBINX, and **E** Fusarium Head Blight Severity FHBSEV

Due to the importance of incorporation of the D genome into SHWs for increasing tolerance to environmental stresses, we also generated Manhattan plots showing MTAs on the D genome (Fig. 5 and Additional Fig. 5). For DON content, we found one MTA on chromosome 5D. For percentage of FDKs, we found two MTAs on chromosome 3D and three MTAs on chromosome 7D. For FHB incidence, we found one MTA on chromosome 1D, one MTA on chromosome 3D, and one MTA on chromosome 7D. For the FHB index, we found one MTA on chromosome 3D and one MTA on chromosome 7D. For FHB severity, we found one MTA on chromosome 3D, three MTAs on chromosome 5D, and two MTAs on chromosome 7D. Individual allele effects for markers associated with FHB traits ranged from a 0.69% decrease in FDK in 2019 to a 71.54% decrease in DON content in 2017. These traits were related to the SNP markers TA002671-0128-w (2B) and Excalibur_rep_c105343_349 (2B), respectively. Other MTAs ranged between 0.69% and 71.54%. For example, the SNP marker CAP12_c731_102 (6B) was associated with an 18% decrease in DON content (Table 2 and Additional Table 1).

**Table 2:** Marker-FHB trait associations, allele effect, and a list of candidate genes that may be involved in FHB resistance (reference genome: IWGSC RefSeq v1.0)

**FHB trait**	**SNP marker**	**Ch**	**Position**	**%Allele effect**	**Potential gene**	**No. reported QTL **[31]
DON content	wsnp_Ex_c40247_47349166	7A	116,113,239	0.04	*TraesCS7A01G160000*	3
	Excalibur_rep_c105343_349	2B	65,114,016	0.72	*TraesCS2B01G131600LC*	2
	wsnp_Ex_c22683_31887799	3B	7,066,858	0.05	*TraesCS3B01G017100*	26
	Tdurum_contig54548_1924	4B	488,676,151	0.08	*TraesCS4B01G234400*	8
	CAP12_c731_102	6B	715,704,861	0.18	*TraesCS6B01G462400*	New
FDK2017	BobWhite_rep_c64679_73	2B	666,650,024	0.16	*TraesCS2B01G470400*	5
	Tdurum_contig51386_128	3B	750,138,040	0.49	*TraesCS3B01G506600*	New
FDK2019	wsnp_Ex_c1997_3756118	1A	514,137,547	0.13	*TraesCS1A01G323600*	3
	Tdurum_contig10482_110	4A	713,522,996	0.02	*TraesCS4A01G445600*	6
	Tdurum_contig49804_392	3B	4,150,864	0.04	*TraesCS3B01G008000*	21
	RAC875_c62400_639	5B	669,897,740	0.11	*TraesCS5B01G503200*	4
	BobWhite_c36455_205	3D	477,639,282	0.03	*TraesCS3D01G363300*	1
FHB INC2019	wsnp_CAP11_c1815_982483	1A	544,054,450	0.19	*TraesCS1A01G363800*	2
FHBINX2017	RAC875_c52338_1019	5A	86,661,890	0.13	*TraesCS5A01G073900*	5
	Excalibur_rep_c103629_346	5A	390,366,984	0.03	*TraesCS5A01G188700*	2
	BS00082460_51	6A	615,589,117	0.01	*TraesCS6A01G416700*	5
	Kukri_c9150_1181	6B	653,221,775	0.05	*TraesCS6B01G378600*	5
FHBINX2019	TA002539-0532	2A	598,941,707	0.27	*TraesCS2A01G509100LC*	6
	Excalibur_c17050_570	6A	581,484,720	0.09	*TraesCS6A01G348800*	1
	wsnp_Ra_c28444_37905400	2B	383,373,141	0.13	*TraesCS2B01G277400*	4
FHBSEV2017	wsnp_Ku_c25809_35776454	3A	525,201,271	0.17	*TraesCS3A01G422800LC*	2
	Excalibur_c8522_1894	7A	4,897,280	0.09	*TraesCS7A01G010800*	New
	GENE-4440_719	7A	14,734,369	0.09	*TraesCS7A01G033500*	2
	wsnp_RFL_Contig3951_4390396	1B	646,167,728	0.16	*TraesCS1B01G423500*	3
	IAAV4252	5B	65,243,608	0.02	*TraesCS5B01G059200*	2
	BS00034658_51	5B	21,006	0.11	*TraesCS5B01G000100*	New
FHBSEV2019	BS00024548_51	3A	700,564,359	0.19	*TraesCS3A01G466700*	1
	wsnp_Ku_c2376_4562448	7A	67,985,152	0.07	*TraesCS7A01G110700*	1
	wsnp_Ra_c28444_37905400	2B	383,373,141	0.10	*TraesCS2B01G277400*	3
	GENE-0293_346	3B	769,345,579	0.07	*TraesCS3B01G779100LC*	New

### Functional characteristics of candidate FHB resistance genes

In total, 395 candidate genes for FHB resistance were located within a 100 kbp region on either side of the peak markers. This interval has been selected based on the rate of LD decay (r^2^ < 0.5) in the current population. While 61% of the candidate genes were involved in a broad range of physiological processes including defense response, the remaining candidate genes (39%) had unknown functions. In the group of candidate genes with identified functions, reverse transcriptases and zinc ion binding proteins had the highest frequency (4%), while protein kinase genes and genes encoding for protein binding had the next highest frequencies (3%). The P-loop containing nucleoside triphosphate hydrolases occurred with a frequency of 2%. Genes encoding HSP40/DnaJ peptide-binding, hydrolase, nucleic binding, pectinesterase inhibitor, protein dimerization, protein transporter, structural constituent of ribosome, sucrose transmembrane transporter, and transferases had frequencies of 1%. Some of the notable candidate genes with frequencies of less than 1% included gibberellin-regulated protein, photosystem II protein, and the stress-induced protein Di19. In particular, SNPs linked to 9 genes on chromosome 2B were associated with both disease severity and disease index in 2019 (Additional Table 1).

## Discussion

The narrow genetic diversity in the wheat gene pool poses major challenges to breeding new wheat varieties with desirable traits. The production of SHWs from crosses between modern durum wheat and its wild relative (*A. tauschii*) can introduce new sources of genetic resistance against abiotic and biotic stresses into accessible germplasm for the wheat breeding program. In general, it offers opportunities to broaden the wheat gene pool.

In the current study, a collection of 194 SHDW lines was used to test FHB resistance and post-harvest traits under inoculated mist-irrigated field conditions in Ontario, Canada over the course of three years (2017–2019). In 2018, FHB pressure was low due to low precipitation. In 2017 and 2019, however, the SHDW lines exhibited significant variations in FHB incidence and severity. The variability of the responses of these inbred lines from one year reflected the variability in the establishment of the FHB disease conditions [32] from year to year. This highlights the need for multi-year testing to evaluate FHB resistance traits.

Results from the current study found that the panel of 194 SHDW lines consisted of three sub-panels. This differed from a previous analysis of the same materials that suggested five subgroups [33]. The discrepancies between the present and the previous study [33] may be due to differences in the number of SNPs (31 K versus 6.904 K) and the types of analyses (fastStructure versus Structure v.2.3.4), respectively. Recently, a principal component analysis (PCA) of 139 winter and spring SHWs was performed using 35,939 high-quality SNPs. This analysis found two subgroups, which were mainly separated by the geographical origin of the durum parents and the growth habit (spring versus winter) of the crop [34]. The current panel includes SHDW lines that were randomly selected from the CIMMYT lines. These lines were derived from crosses between 19 *Ae. tauchii* and 13 modern tetraploid wheat parents, and later were backcrossed onto adapted hexaploid lines. The previous study [33] revealed that the D genome made a greater contribution to diversity (*R*^*2*^ = 3.48) than the tetraploid parents (*R*^*2*^ = 2.75). In this study, the genetic structure of the SHDW was not found to be related to the origin of *Ae.tauschii* or the tetraploid parents.

Our results identified marker-trait associations with resistant allele effects between 0.69%-71.54% for FHB field and post-harvest traits. This is an important resource for FHB resistance marker development, which improves the efficiency of selection for FHB resistance traits for variety development [34]. In line with a previous study [35], no overlapping QTL were detected on chromosomes 7A, 2B, 3B, 4B, and 6B for DON content, FHB incidence, or FHB severity [35]. QTL involved in DON content may act independently of those related to FHB field resistance components (e.g. incidence, severity, and index). Our data suggest that this is similarly the case in the SHDW panel. To breed for DON reduction in the grain, the QTL involved in DON suppression can be introduced into wheat lines independent of the QTL involved in the other FHB-resistant components.

The D genomes of disparate SHW populations display higher nucleotide sequence diversity compared with the D genome of bread wheat [34, 36]. In a recent study of 101 SHW lines, 35,939 SNPs were equally distributed among A, B, and D chromosomes (33%, 36%, and 31%, respectively) [34]. This is inconsistent with the lower numbers of SNPs on D chromosomes (14%) relative to A (38%) and B (48%) chromosomes in our SHDW panel. Despite these differences, 4 regions on chromosomes 7D, 3D, and 5D were associated with FDK, FHB index, and FHB severity. In the FHB-resistant hexaploid Sumai 3 [37], the D genome is not involved in FHB resistance [38]. These results are consistent with the view that the utilization of SHDW lines in wheat breeding programs might add new sources of FHB resistance to the narrow gene pool of hexaploid wheat germplasm. Furthermore, two QTL *(QFhb.hbaas-2DS* and *QFhb.hbaas-4DS*) that decreased FHB severity by 69.9% and 55.5%, respectively, were identified in a doubled haploid population of a cross between moderately resistant Jingzhu 66 and susceptible Aikang 58 [39]. In addition, a recent study suggested that the incorporation of SHW populations in the CIMMYT wheat breeding program contributed significantly to the D genome diversity (15.6%) and yield in international yield trials (20%). This is further evidence that SHW lines can increase genetic diversity in the wheat gene pool [15].

In this study, we detected 395 candidate genes in regions spanning 100 kbp on both sides of the SNP markers associated with FHB resistance traits. These marker-trait associations during our three-year experiments included 56 genes for DON (2017), 19 for FDK (2017), 59 for FDK (2019), 28 for FHB incidence (2019), 33 for FHB index (2017), 38 for FHB index (2019), 95 for FHB severity (2017), and 67 for FHB severity (2019) (Additional Table 1). Genes involved in DON detoxification such as glycosyltransferases play important roles in FHB resistance [40, 41]. The importance of glycosyltransferases is exemplified by the upregulation of 69% of 179 UDP-glycosyltransferases (UGT; on chromosomes A, B, and D) four days after inoculation of wheat heads with a DON-producing *F. graminearum* isolate [40]. Further, overexpression of *TaUGTs* in a susceptible line (Fielder) resulted in a lower DON content than the wild type [42]. Notably, our results identified 4 UGTs (*TraesCS3B01G017200*, *TraesCS7D01G117800*, *TraesCS4A01G445700*, and *TraesCS5B01G059400*) in the SHDW panel (Additional Table 1). Interestingly, the SNP marker CAP12_c701_102 associated with an 18.1% effect on DON reduction in *TraesCS6B01G462400*, belongs to the group of proteins with gibberellic acid-stimulated regulatory function involved in diverse processes. These processes include wounding and pathogen infection stresses [43, 44], and regulation of flowering time [45-47]. We also detected *TraesCS3B01G017100* with an ABC transporter function related to DON content and *TraesCS7A01G449500*, *TraesCS7A01G449600*, *TraesCS6B01G407000*, and *TraesCS6B01G407100* with cytochrome P450 activity related to FDK and FHB incidence. This is consistent with increased transcript levels of genes encoding ABC transporters, UGTs, cytochrome P450s (cytP450s), and glutathione-S-transferases in DON-treated barley spikes [48]. In plants, cytP450 mono-oxygenases metabolize a large number of different substrates in biosynthetic and detoxification pathways. The metabolic products of cytP450s play important roles in plant defense response and display antifungal activity [49]. Specifically, the resistant responses of wheat leaf and spike to artificial inoculation with *F*. *graminearum* spores and DON treatment were accompanied by the upregulation of cytP450s [50].

In this study, 100 kbp distance from the SNP marker on both sides was used to detect the potential candidate genes involved in the FHB resistance. This region could be expanded to detect important potential candidate genes. Therefore, further validation is necessary to substantiate the potential involvement of the detected genes in FHB resistance based on the observed results.

Until 2009, approximately, 100 QTL associated with FHB resistance have been mapped to wheat chromosomes except for chromosome 7D [51]. In a population resulting from a cross between two moderately resistant Chinese wheat cultivars, Zhengmai 9023 and Yangmai 158, one QTL from Zhengmai 9023 located on 7D explained 6.15% to 9.32% of the phenotypic variations [52]. A QTL with a minor effect (5.6 ~ 7.5%) was also mapped on 7D. This QTL contributed to type II resistance in a population that resulted from a cross between Haiyanzhong and Wheaton. This QTL was previously reported in Arina and Wangshuibai [53]. We identified *TraesCS7D01G411600* on 7D, which encodes a 60S acidic ribosomal protein. It has been reported that DON inhibits protein and nucleic acid biosynthesis by binding to the 60S ribosome subunit [54]. It has been suggested that the 60S ribosomal protein interacts with peptide elongation factors during protein synthesis [55]. The candidate gene, *TraesCS7D01G411600* may play a role in DON activity on the 60S ribosome subunit. In addition, the SNP marker associated with *TraesCS7D01G411600* could be used to screen wheat germplasm for lines with alleles for resistance against DON. Another candidate gene *TraesCS7D01G411700* encodes a knottin, scorpion toxin-like protein. This protein interacts with phospholipids and sphingolipids of fungal membranes [56] and has antimicrobial activity [57].

The products of genes encoding pectin esterase inhibitors act against the polygalacturonase activity of Fusarium [58]. In durum wheat, ectopic expression of a pectin methyl esterase inhibitor (PMEI), which regulates pectin methyl esterase (PME) activity, resulted in increased resistance to both FHB and spot blotch (*Bipolaris sorokiniana*) [59]. Therefore, 3 PMEI encoding candidate genes in the SHDW panel, *TraesCS3B01G008200*, *TraesCS3B01G008300*, and *TraesCS3B01G00840*0 (Additional Tables 1 and 2) may play roles in regulating *F. graminearum* PME activity, which has been shown to enhance fungal colonization and virulence on wheat spikes [60].

In the SHDW panel, a SWEET gene *TraesCS7A01G159800* may constitute part of a defense mechanism to restrict sugar availability and proliferation of *F. graminearum* but may equally be important for other known developmental processes including glucose efflux from the tapetum for pollen growth [61]. The SWEET class of sugar efflux carriers is involved in sugar diffusion across cell membranes [61, 62]. Overexpression of SWEET10 in sweet potatoes decreased soluble sugars and increased resistance to *F. oxysporum* [63].

Lectins, including LRR lectins, are carbohydrate-binding proteins involved in defense against insects as well as viral, bacterial, and fungal pathogens [64]. Several lectin family proteins were upregulated in genotype-specific manners following inoculation of wheat with *F. graminearum* [65]. It is intriguing to speculate that *TraesCS7A01G634900LC*, which encodes a TRAF-like protein (Additional Table 1), may have lectinic activity and play a role in defense against* F*. *graminearum*. Also, jacalin-related lectins (JRL) are prominent plant defense-related lectins that are associated with disease resistance, abiotic stress signaling, wounding, and insect damage [66, 67]. For example, the mannose-specific wheat lectin TaJRLL1 is mainly expressed in stems and spikes and is involved in a resistance response against *F*. *graminearum* [68]. Further, the chimeric lectin encoded by wheat *Fhb1* is a major genetic determinant of FHB resistance [69]. The significance of lectins in FHB resistance is further emphasized in the present study by the association of 4 Jacalin-like lectins and a Kelch-type beta-propeller (a chimeric JRL) with FHB traits (Additional Table 1).

An LSM domain-containing proteins (with an FDF domain of unknown role) are part of a complex in the mRNA de-capping machinery [70]. In Arabidopsis, the cytoplasmic LSM proteins are major regulators of abiotic stress responses including low temperature, salt, and drought stresses [71]. In addition, they regulate plant adaptation responses to adverse environmental conditions through stress-dependent regulation of mRNA turnover by targeting selected stress-inducible transcripts (LEA7, ZAT12, ABR1, ANAC019, AHK5, or ANAC092) for de-capping and degradation [71]. In our study, an LSM domain-containing protein and a late embryogenesis abundant protein (LEA-14) were associated with FHB resistance (Additional Table 1) suggesting that the LSM domain-containing proteins and their targets may also be involved in the regulation of plant biotic stress responses in the SHDW panel.

Based on the comparison of genomic regions associated with FHB resistance in the current study and previously reported QTL, seven unique genomic regions were identified. These regions include two genic regions on 3B for FHB severity and FDK, respectively; two regions on 5B for FHB severity; one region on 7A for FHB severity, and one region on 7D for FDK. The specific details of these unique genomic regions are shown in Table 2 and Additional Table 1. The comparison was conducted using data from Zheng et al. [31] and WheatMine (IWGSC RefSeq v1.0 assembly), which contained information on 625 QTL from 113 publications.

In this study, 7% of the significant marker-trait associations were located on chromosome D which was less than the contribution of chromosomes A and B. While the D genome does contribute to genetic diversity in wheat, our study showed that it does not provide significant resistance to Fusarium head blight (FHB). Instead, resistance to FHB is largely attributed to genes present in the A and B genomes in the SHDW panel. Therefore, breeding programs focused on developing FHB-resistant wheat varieties typically prioritize genes found in the A and B genomes over those found in the D genome. However, it is important to note that the D genome can still contribute to other desirable traits in wheat, such as drought tolerance [72], disease resistance to other pathogens [73], and improved grain quality [74]. Therefore, the presence of the D genome derived from the wild species in wheat can still be beneficial for overall crop improvement efforts.

## Conclusions

This study provides new insights into the genetic basis of FHB resistance in SHDW lines. Candidate genes encoding lectins, ABC transporters, cytP450, UGTs, knottin, 60S acidic ribosomal protein, and LSM domain-containing proteins may be involved in a defense network to suppress *F*. *graminearum* growth and DON production. Once validated, the markers associated with FHB resistance traits can be utilized for DNA marker-assisted selection. The incorporation of SHDW lines into wheat breeding schemes will offer a novel approach for the introgression of disease resistance into the conventional wheat gene pool and may mitigate the impact of FHB on wheat production.

## Methods

### Plant materials and field experiments

A set of 200 spring wheat lines consisting of 194 accessions of spring Synthetic Hexaploid Derived Wheat (SHDW) from CIMMYT and six check cultivars were planted for three years (2017–2019) in two replications in an FHB nursery at the Elora research station (University of Guelph, Canada). The check cultivars consisted of Sable (highly susceptible), Norwell (susceptible), Carberry (moderately susceptible), AAC Scotia (moderately resistant), Hoffman (susceptible), and Pasteur (susceptible). The SHDW panel was derived from crosses between 19 *Ae. tauschii* and 13 tetraploid accessions with synthetic degrees of 2–5 [33]. Genetically fixed SHW lines were later crossed with one or four adapted hexaploid wheat lines resulting in 2^nd^- and 5^th^-degree synthetic hexaploid-derived wheat lines [33]. The experimental design was a randomized complete block design (RCBD). For each line, 100 seeds were planted in a one-metre row with a row spacing of 38 cm. After planting, the plots were fertilized with urea (NPK 46–0-0; 70 kg nitrogen/ha). Weeds were controlled by manual and mechanical weeding. Wheat spikes were harvested manually to prevent Fusarium Damaged Kernel (FDK) loss and were threshed using a belt thresher (ALMACO, IL, USA).

### *F. graminearum* inoculum preparation and field inoculation

A mixture of three *F. graminearum* isolates from Ontario, Canada, consisting of 3ADON, 15ADON, and an undetermined chemotype was used for field inoculations. The *F. graminearum* inoculum was prepared for artificial inoculation in the field as described previously [75]. A 0.5 cm^2^ disc of *F. graminearum* mycelium on potato dextrose agar (PDA) was cut and transferred to a sterilized medium consisting of five g of chopped wheat straw in 125 ml water. The inoculated medium was placed on a shaker and grown for 14 days at 120 rpm in the dark at 25 °C. The macroconidia were harvested and counted using a hemocytometer (Sigma-Aldrich, Oakville, ON, Canada). Before dusk, wheat plants were sprayed three times at two days pre-anthesis, anthesis, and two days post-anthesis with the *F. graminearum* spore suspension (50,000 macroconidia ml^−1^). Plots were mist irrigated (1–2 h/day) to generate approximately 70% relative humidity across the FHB nursery.

### Phenotypic evaluation

FHB incidence and severity were evaluated 21 days post-inoculation. In each plot, FHB incidence was determined based on the number of infected wheat heads in 100 heads. FHB severity was evaluated based on the disease progress in each wheat head (0–100%) as described previously [76]. The disease index was calculated as follows.

$$\mathrm{Disease\,index}=\frac{\mathrm{FHB\,incidence }\times \mathrm{ FHB\,severity}}{100}$$

For each wheat line, FDK (%) was determined in a sample of 100 seeds in two replications. For DON measurement, a five g seed sample was ground to a fine powder, and DON was extracted and quantified by a Neogen Veratox 5/5 ELISA kit (MI, USA) according to the manufacturer’s instructions. Phenotypic data (FHB incidence, severity, index, %FDK, and DON content) were analyzed using PROC MIXED (V 9.4, SAS Institute Inc., Cary, NC, USA) with block as random and year and genotypes as fixed effects. Normality was tested using a Shapiro-Wilks test in the PROC UNIVARIATE. For principal component analysis (PCA), PROC PRINQUAL was conducted to produce bi-plots. PROC CORR was used to create a correlation table. For correlation matrix analysis, the PerformanceAnalytics package was used in RStudio.

### Genotypic evaluation

DNA was extracted using DNeasy Plant Mini Kit (Qiagen, Hilden, Germany) according to the manufacturer’s instructions. All accessions were genotyped using Illumina’s iSelect 90 K SNP chip [77] at the National Research Council of Canada in Saskatoon, Saskatchewan, Canada [33]. Genotypic data were re-analyzed and filtered out for missing data > 10%, minor allele frequency (MAF) < 5%, and heterozygosity > 50%. The imputation of missing data was performed with BEAGLE v5.1 [78, 79].

### Population genetic analysis

Population structure was estimated using fastStructure [27]. Five runs were performed for each number of populations (K) set from 1 to 12. Then, a ChooseK analysis was conducted to determine the number of subpopulations. A principal component analysis (PCA) [80] was conducted in PLINK [81]. A neighbor-joining phylogenetic tree [28] was constructed in MEGA7 [29]. The taxa were clustered, and the reliability of these clusters was assessed by bootstrapping (1,000 replicates) [30]. Genome-wide pairwise linkage disequilibrium (LD) analysis (r^2^ and D´) was performed using all SNPs and LD decay was calculated using PopLDdecay [82].

### Genome-wide association analysis

GWAS was conducted using the rMVP package in R [83] utilizing Fixed and random model Circulating Probability Unification (FarmCPU) model [84]. The PCA (covariate P) and kinship (covariate K; calculated by FarmCPU) were used in the model to capture panel structure and relatedness among individuals, respectively [85–87]. To ensure a false discovery rate (FDR) < 0.1, an adjusted *p*-value (*q* value) was used to establish a significance threshold [77]. The *p*-value distributions of markers (observed *p*-values plotted against expected *p*-values) were calculated in Q-Q plots. Manhattan and Q-Q plots were drawn using CMplot. The WheatMine database (Wheat IWGSC RefSeq v1.0 data) was used to identify candidate genes associated with the SNP markers within a region extending to 100 kbp at either side of the peak marker. The allele effect of each QTL was calculated based on the average of each FHB trait.

## Supplementary Information

**Additional file 1: Additional Figure 1.** Correlations among FHB resistance traits in SHDW lines in 2017 (A), 2018 (B), and 2019 (C).

**Additional file 2: Additional Figure 2.** A principal component analysis (PCA) [80] performed in PLINK [81] shows the positions of the individuals in the three subpopulations; PCA1=52.7%; PCA2=30.6%;PCA3=16.5%.

**Additional file 3: Additional Figure 3.** LD Decay.

**Additional file 4: Additional Figure 4.** Manhattan plots of associations between SNPs and FHB traits in the SHDW panel across three years (2017-19). A) Deoxynivalenol content (DON ppm), B) The average of Fusarium Damaged Kernels (FDKave), C) Fusarium Head Blight Incidence (FHBINC), D) Fusarium Head Blight Index (FHBINX), and E) Fusarium Head Blight Severity (FHBSEV).

**Additional file 5: Additional Figure 5.** Q-Q plots of expected and observed associations between polymorphic SNPs and FHB traits across three years (2017-2019).

**Additional file 6: Additional Table 1.** Marker-trait associations for the FHB resistance traits and selected genes associated with the QTL identified in the present study.

**Additional file 7: Additional Table 2.** Eleven accessions displayed < 10 ppm DON in 2017 and 2019.

## Data Availability

This article represents all data including the additional information that was generated and analyzed during the experimental period of 2017–2019. The phenotypic and SNP associations with FHB traits data in this study were deposited in the Figshare database and are accessible at the following links. https://doi.org/10.6084/m9.figshare.21904845.v1 [88] and https://doi.org/10.6084/m9.figshare.21904890.v2) [89].

## Abbreviations

ABC: ATP-binding cassette
CIMMYT: International Maize and Wheat Improvement Center
DON: Deoxynivalenol
FDKs: Fusarium damaged kernels
FHB: Fusarium head blight
GWAS: Genome wide association study
INC: Incidence
INX: Index
JRL: Jacalin-related lectins
Kbp: Kilobase pair
LD: Linkage disequilibrium
LRR: Leucine-rich repeats
MAF: Minor allele frequency
MTAs: Marker-trait associations
PCA: Principle component analysis
PME: Pectin methyl esterase
q-q: Quantile–quantile
QTL: Quantitative trait loci
SEV: Severity
SHDW: Synthetic hexaploid derived wheat
SNP: Single nucleotide polymorphism
SSR: Single nucleotide repeat
STS: Sequence-tagged site
SWEET: Sugars will eventually be exported transporter
UGT: UDP-glycosyltransferases
