# Supplementary figures and images for: Identification of fusarium head blight resistance markers in a genome-wide association study of CIMMYT spring synthetic hexaploid derived wheat lines
Source: BMC Plant Biol. 2023 May 31;23:290. doi: 10.1186/s12870-023-04306-8 (PMC10230752; doi:10.1186/s12870-023-04306-8)

## Slide 1
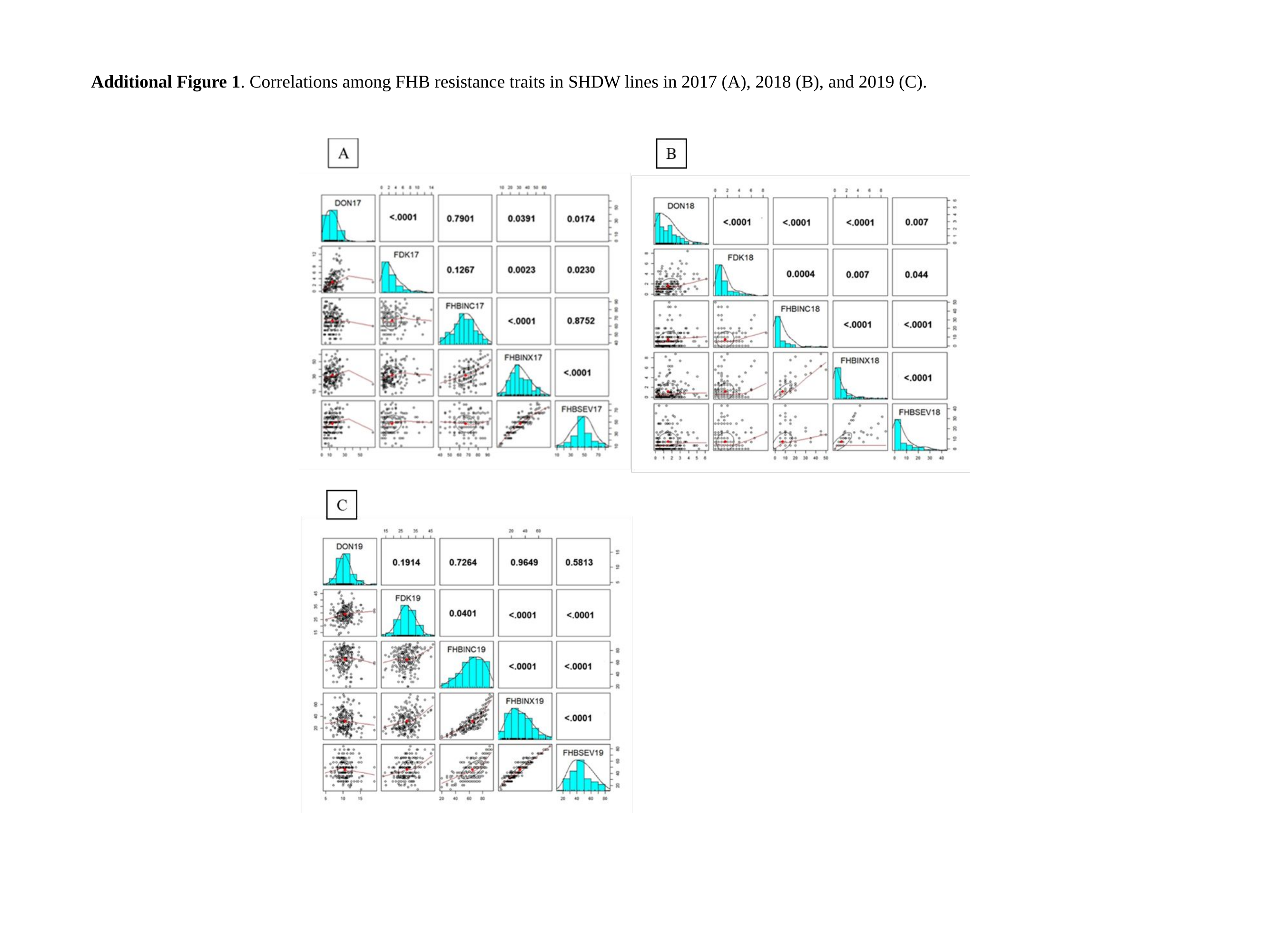

Additional Figure 1. Correlations among FHB resistance traits in SHDW lines in 2017 (A), 2018 (B), and 2019 (C).

Supplement: Supplementary file 1 — Additional file 1: Additional Figure 1. Correlations among FHB resistance traits in SHDW lines in 2017 (A), 2018 (B), and 2019 (C). [file 12870_2023_4306_MOESM1_ESM.pptx]

## Slide 1
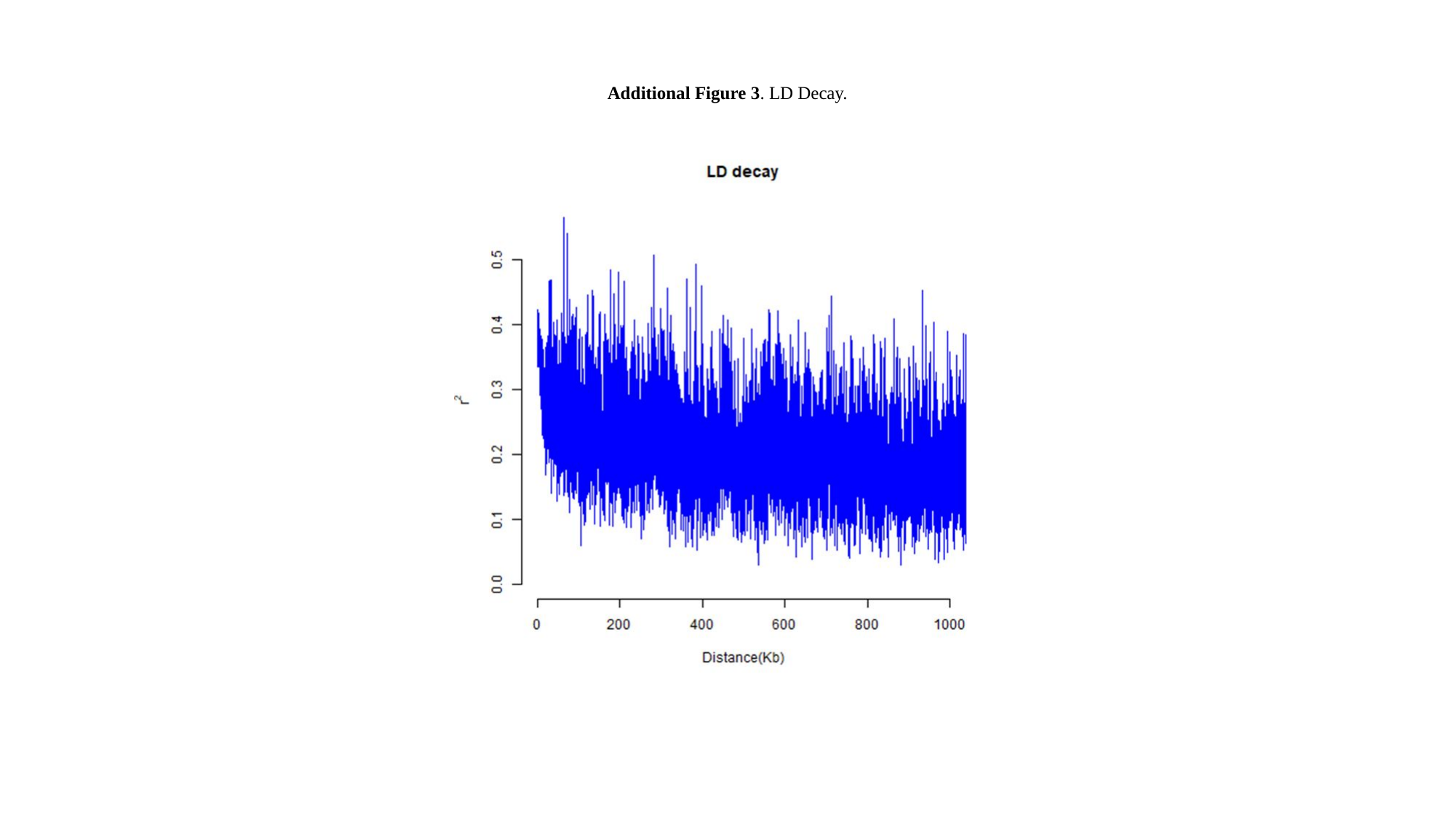

Additional Figure 3. LD Decay.

Supplement: Supplementary file 3 — Additional file 3: Additional Figure 3. LD Decay. [file 12870_2023_4306_MOESM3_ESM.pptx]
