# Supplementary material for: Identification of fusarium head blight resistance markers in a genome-wide association study of CIMMYT spring synthetic hexaploid derived wheat lines
Source: BMC Plant Biol. 2023 May 31;23:290. doi: 10.1186/s12870-023-04306-8 (PMC10230752; doi:10.1186/s12870-023-04306-8)
Supplement: Supplementary file 2 — Additional file 2: Additional Figure 2. A principal component analysis (PCA) [80] performed in PLINK [81] shows the positions of the individuals in the three subpopulations; PCA1=52.7%; PCA2=30.6%;PCA3=16.5%. [file 12870_2023_4306_MOESM2_ESM.pptx]

## Slide 1
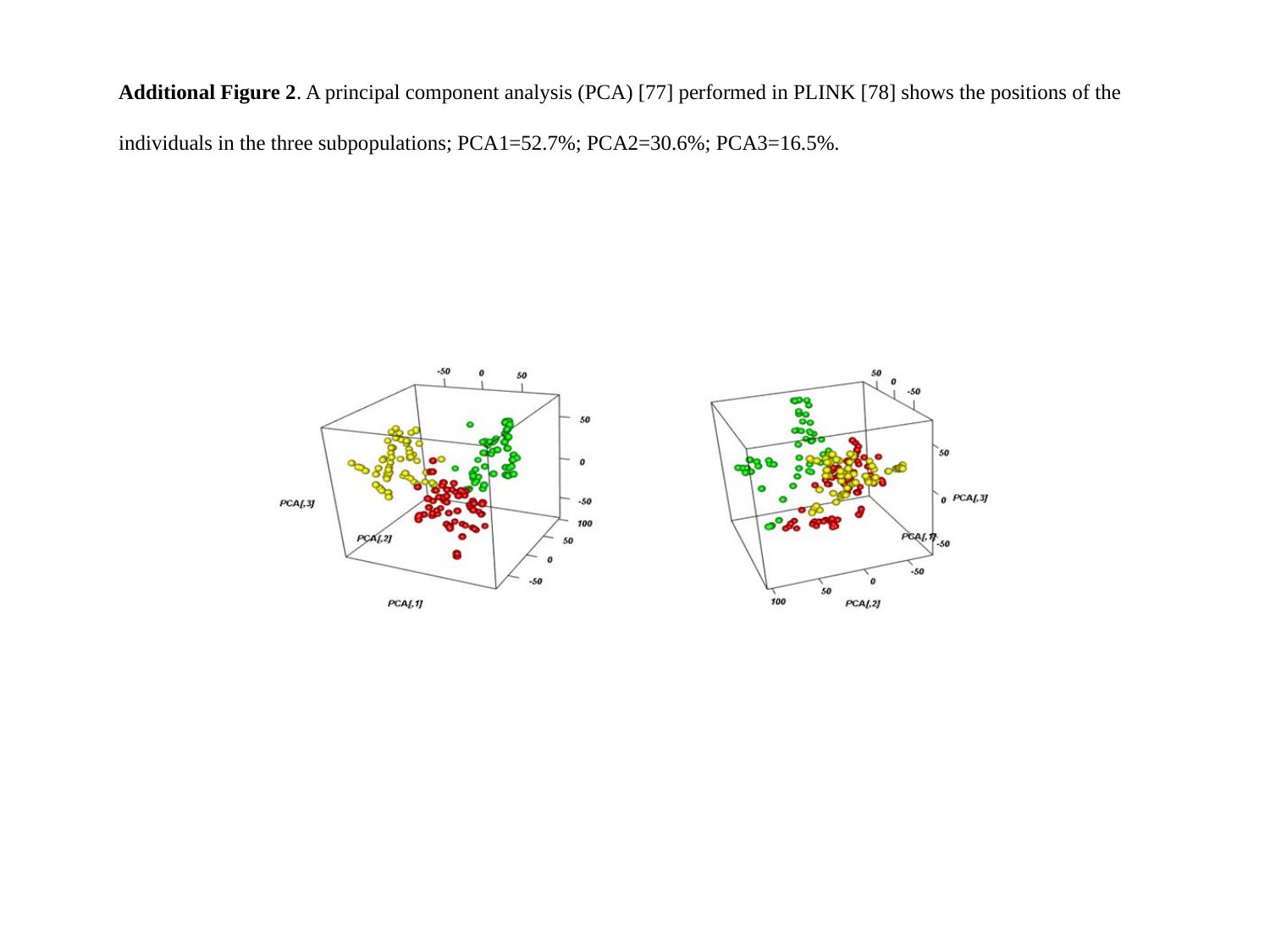

Additional Figure 2. A principal component analysis (PCA) [77] performed in PLINK [78] shows the positions of the individuals in the three subpopulations; PCA1=52.7%; PCA2=30.6%; PCA3=16.5%.
