# Supplementary material for: Identification of fusarium head blight resistance markers in a genome-wide association study of CIMMYT spring synthetic hexaploid derived wheat lines
Source: BMC Plant Biol. 2023 May 31;23:290. doi: 10.1186/s12870-023-04306-8 (PMC10230752; doi:10.1186/s12870-023-04306-8)
Supplement: Supplementary file 4 — Additional file 4: Additional Figure 4. Manhattan plots of associations between SNPs and FHB traits in the SHDW panel across three years (2017-19). A) Deoxynivalenol content (DON ppm), B) The average of Fusarium Damaged Kernels (FDKave), C) Fusarium Head Blight Incidence (FHBINC), D) Fusarium Head Blight Index (FHBINX), and E) Fusarium Head Blight Severity (FHBSEV). [file 12870_2023_4306_MOESM4_ESM.pptx]

## Slide 1
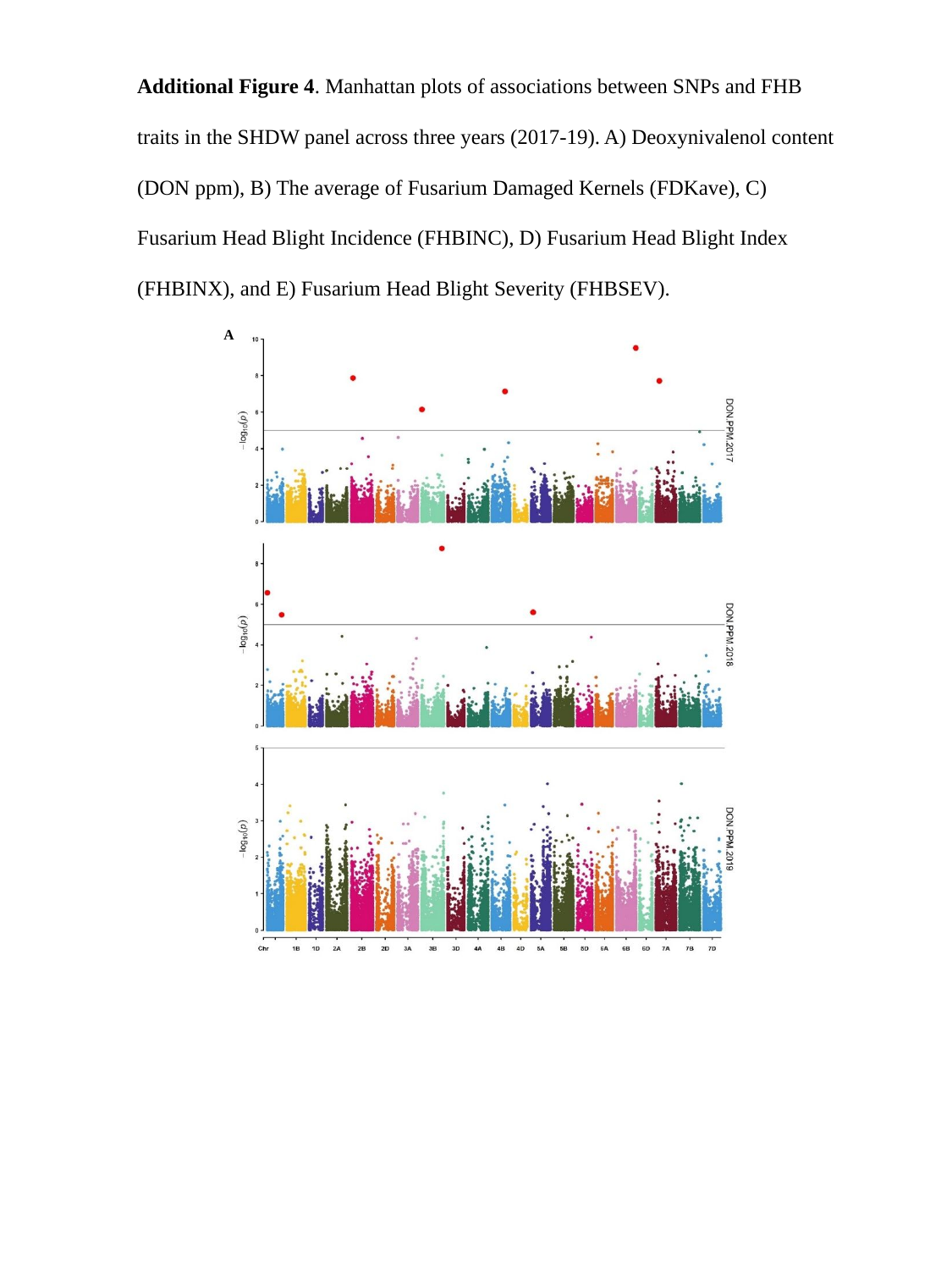

Additional Figure 4. Manhattan plots of associations between SNPs and FHB traits in the SHDW panel across three years (2017-19). A) Deoxynivalenol content (DON ppm), B) The average of Fusarium Damaged Kernels (FDKave), C) Fusarium Head Blight Incidence (FHBINC), D) Fusarium Head Blight Index (FHBINX), and E) Fusarium Head Blight Severity (FHBSEV).
A

## Slide 2
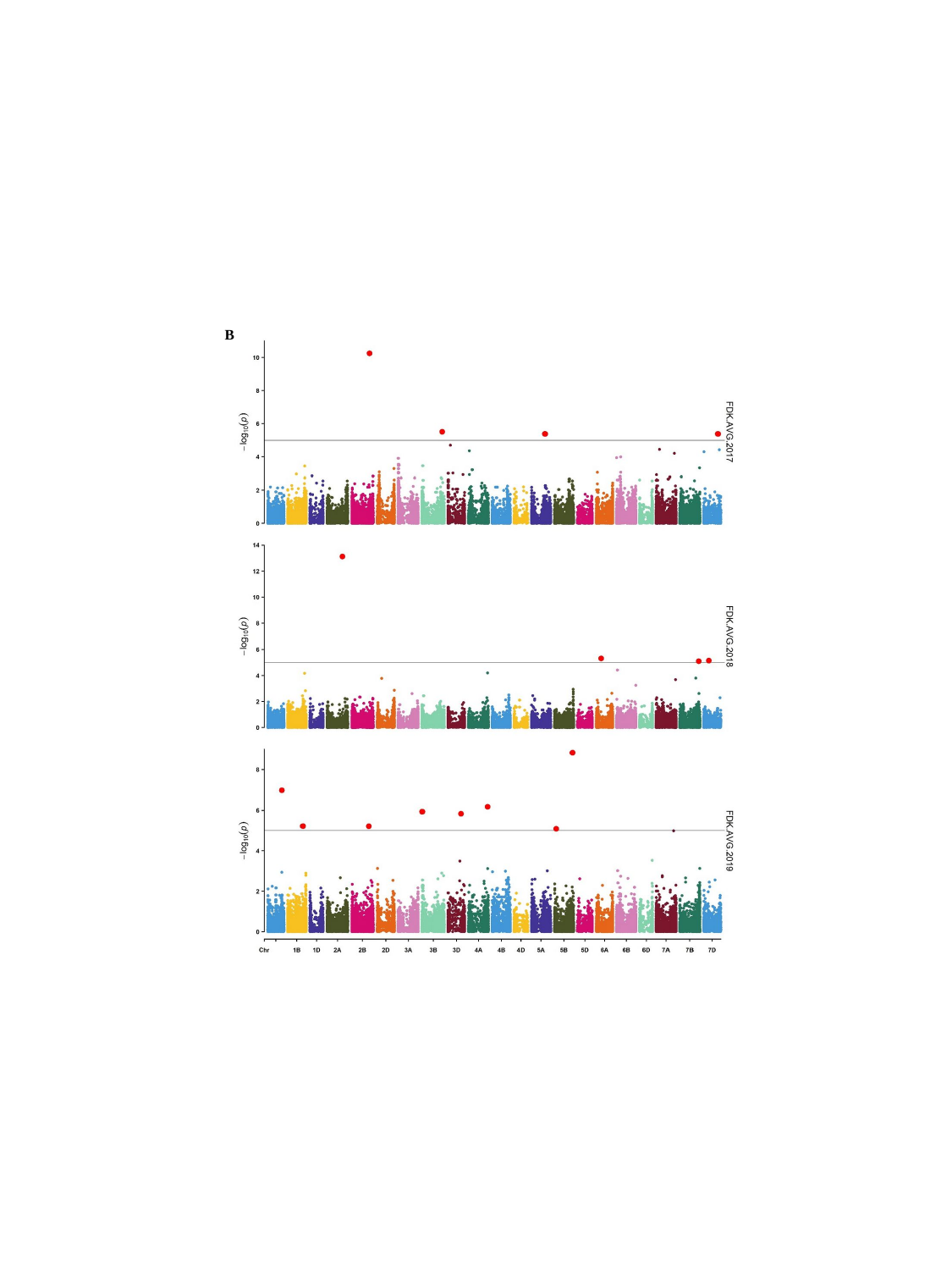

B

## Slide 3
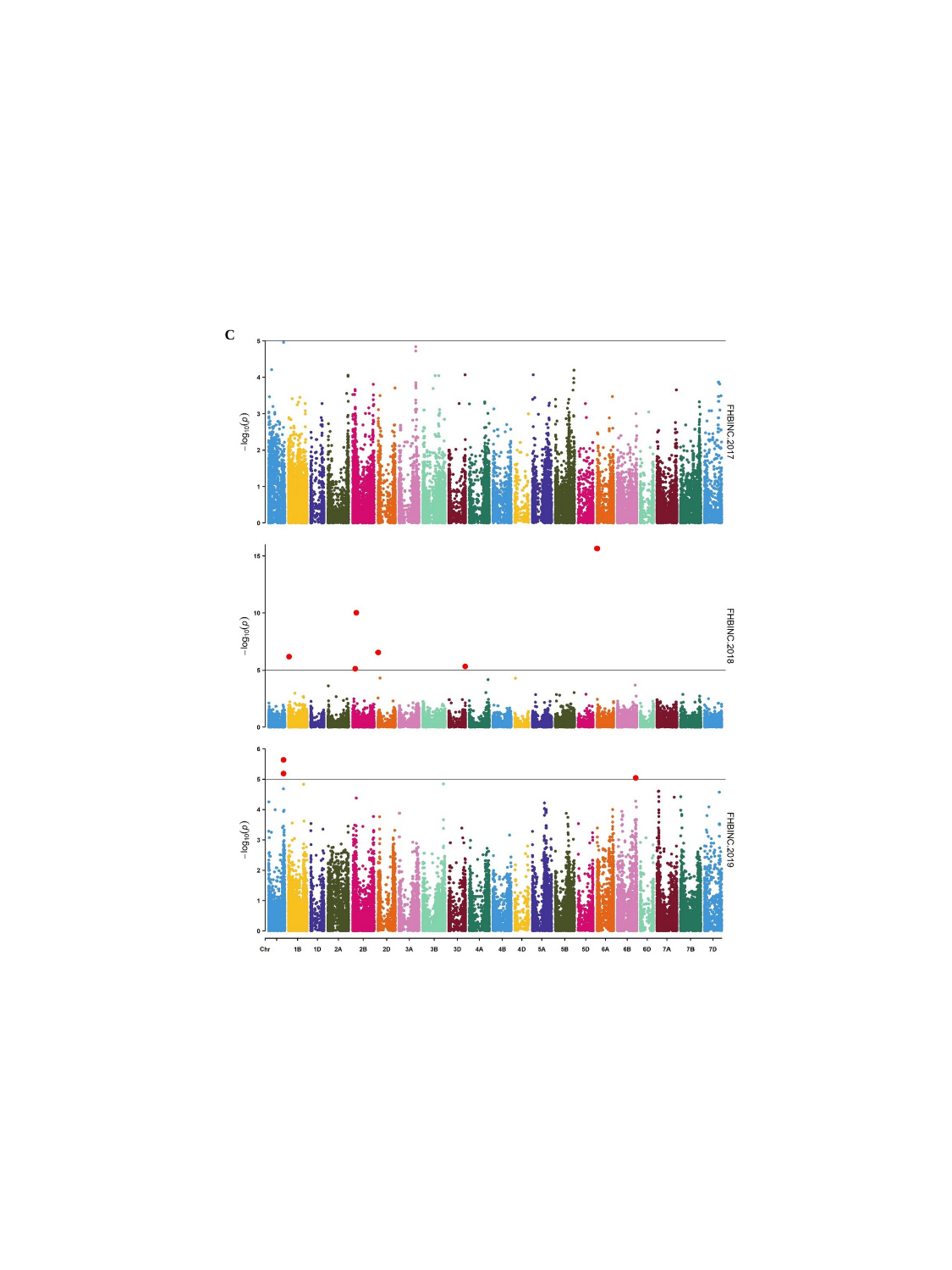

C

## Slide 4
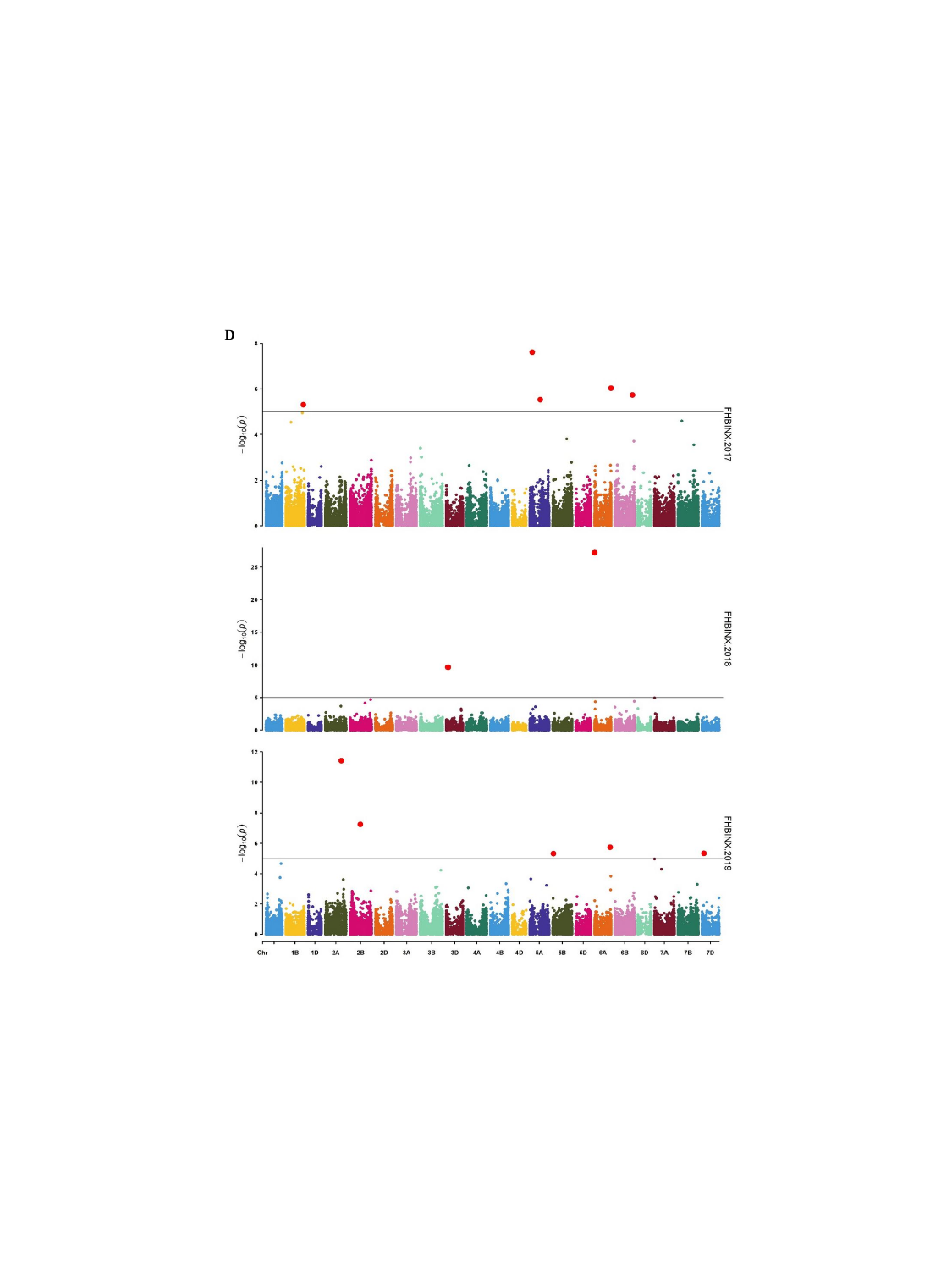

D

## Slide 5
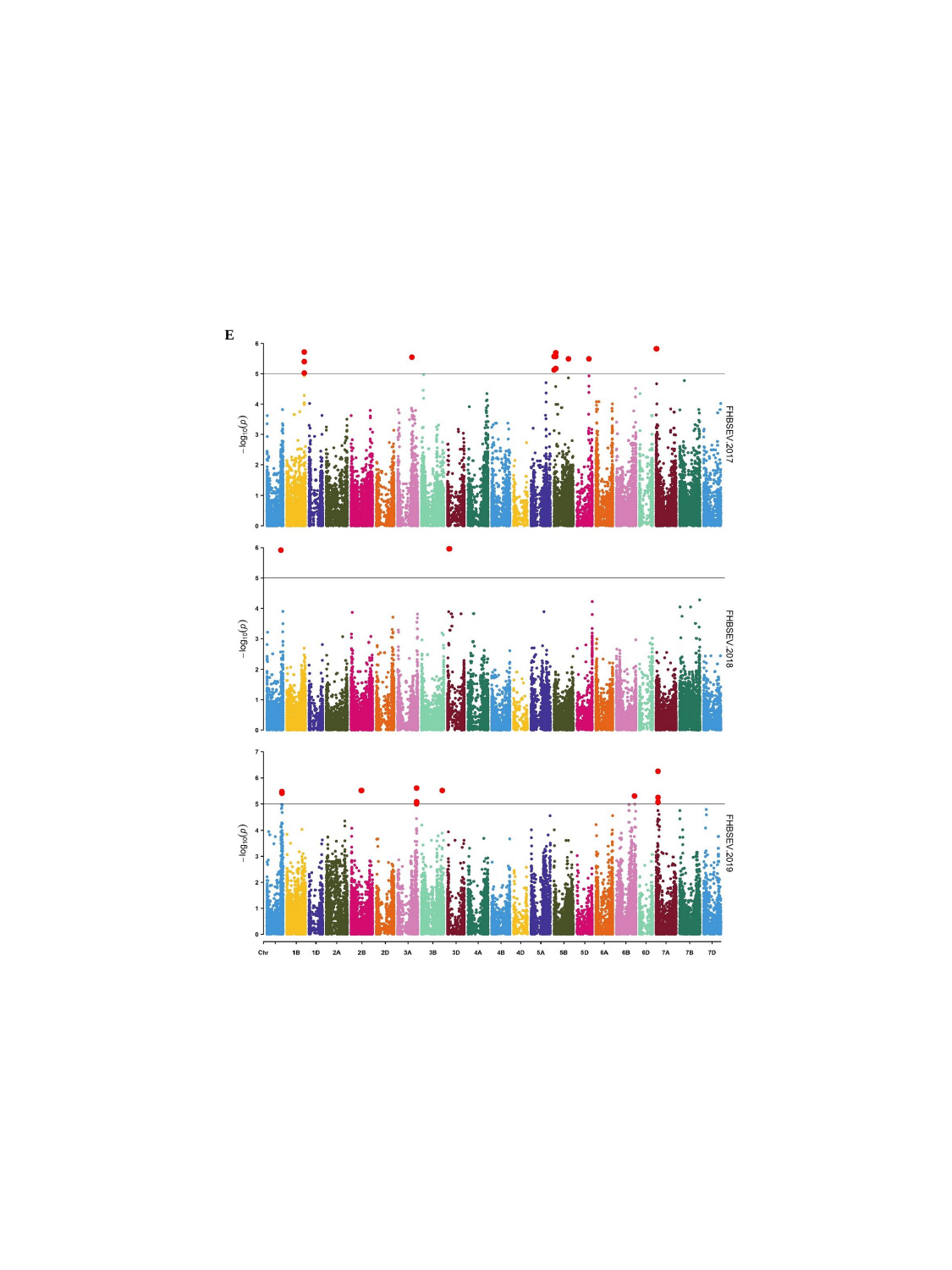

E
