# Supplementary material for: Identification of fusarium head blight resistance markers in a genome-wide association study of CIMMYT spring synthetic hexaploid derived wheat lines
Source: BMC Plant Biol. 2023 May 31;23:290. doi: 10.1186/s12870-023-04306-8 (PMC10230752; doi:10.1186/s12870-023-04306-8)
Supplement: Supplementary file 5 — Additional file 5: Additional Figure 5. Q-Q plots of expected and observed associations between polymorphic SNPs and FHB traits across three years (2017-2019). [file 12870_2023_4306_MOESM5_ESM.pptx]

## Slide 1
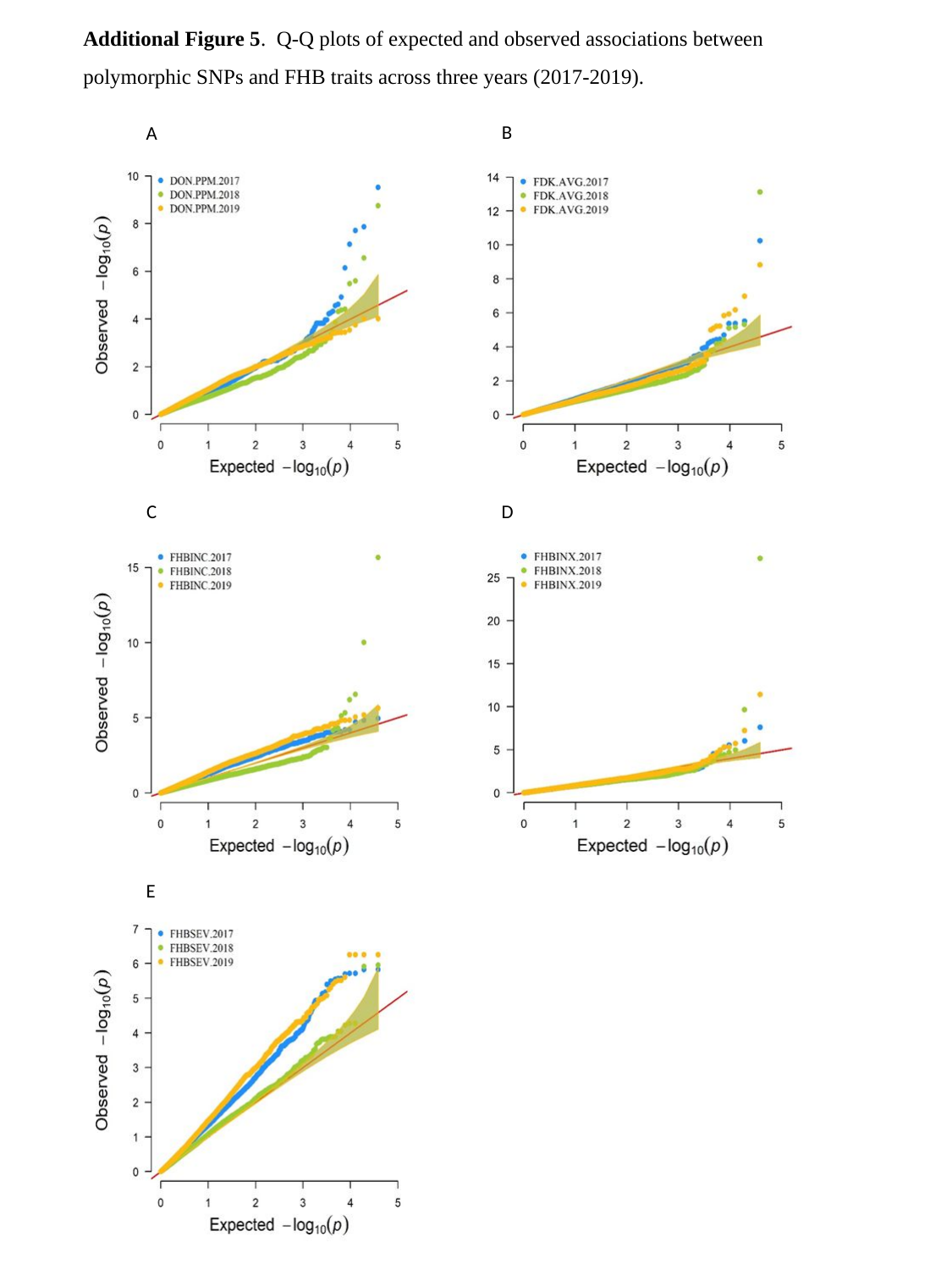

Additional Figure 5. Q-Q plots of expected and observed associations between polymorphic SNPs and FHB traits across three years (2017-2019).
B
A
C
D
E
